# Supplementary material for: Splice-Junction-Based Mapping of Alternative Isoforms in the Human Proteome
Source: Cell Rep. Author manuscript; Available in PMC 2020 Jan 15. (PMC6961840; doi:10.1016/j.celrep.2019.11.026)
Supplement: 2 [file NIHMS1546469-supplement-2.zip › DF1/PXD010154/Liver/Liver_15_MAOB_KIPSDAPWKAPLAEEWDNMTMK.pdf]

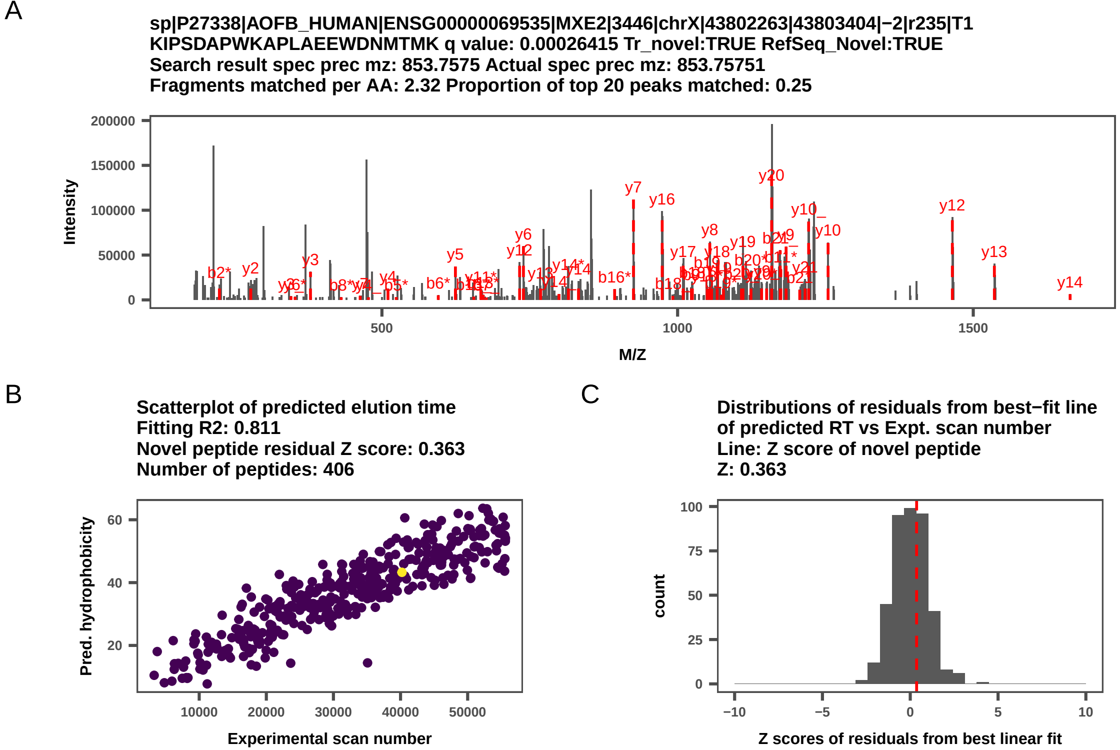

A
sp|P27338|AOFB_HUMAN|ENSG00000069535|MXE2|3446|chrX|43802263|43803404|−2|r235|T1
KIPSDAPWKAPLAEEWDNMTMK q value: 0.00026415 Tr_novel:TRUE RefSeq_Novel:TRUE
Search result spec prec mz: 853.7575 Actual spec prec mz: 853.75751
Fragments matched per AA: 2.32 Proportion of top 20 peaks matched: 0.25
Intensity
200000
150000
100000
50000
0
500
1000
1500
M/Z
y20
y7
y16
y12
y10
y13
y14
y8
y6
y5
y3
y2
b2*
b6*
b16*
y17
y18
y19
B
Scatterplot of predicted elution time
Fitting R2: 0.811
Novel peptide residual Z score: 0.363
Number of peptides: 406
Pred. hydrophobicity
60
40
20
10000
20000
30000
40000
50000
Experimental scan number
C
Distributions of residuals from best−fit line
of predicted RT vs Expt. scan number
Line: Z score of novel peptide
Z: 0.363
count
100
75
50
25
0
−10
−5
0
5
10
Z scores of residuals from best linear fit
